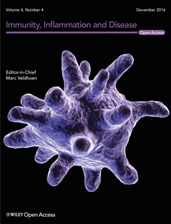# Issue Information

**DOI:** 10.1002/iid3.84

**Published:** 2016-11-28

## Abstract